# Willingness to use a drug consumption room among people who use drugs in Lyon, France, a city with no open scene of drug use (the TRABOUL survey)

**DOI:** 10.1186/s12954-023-00887-7

**Published:** 2023-10-16

**Authors:** Mathieu Chappuy, Philippe Lack, Baptiste David, Gilles Penavayre, Damien Thabourey, Maira Landulpho, Anthony Plasse, Christophe Icard, François Bailly, Faroudja Boutahra, Pierre Pradat, Marianne Maynard, Marie Jauffret-Roustide, Julia de Ternay, Benjamin Rolland

**Affiliations:** 1 Service d’Hépatologie et d’Addictologie, Hôpital de la Croix-Rousse, Hospices Civils de Lyon, Lyon, France; 2 Service Universitaire d’Addictologie de Lyon (SUAL), Hospices Civils de Lyon, Hôpital Edouard Herriot, Pavillon K, 5 Place d’Arsonval, 69002 Lyon, France; 3 Service Universitaire d’Addictologie de Lyon (SUAL), CH Le Vinatier, Bron, France; 4 Service Pharmaceutique, Hospices Civils de Lyon, Lyon, France; 5 Association Aria Oppélia, Villeurbanne, France; 6 CAARUD Pause Diabolo, Association le Mas, Lyon, France; 7 SMPR de Corbas, Pôle SMDPL, CH Le Vinatier, Bron, France; 8 Centre de Recherche Clinique, Hôpital de la Croix Rousse, Hospices Civils de Lyon, Lyon, France; 9 Centre d’Étude Des Mouvements Sociaux (Inserm U1276/CNRS UMR 8044/EHESS), Paris, France; 10 British Columbia Center on Substance Use (BCCSU), British Columbia University, Vancouver, Canada; 11 Baldy Center on Law and Social Policy, Buffalo University, Buffalo, USA; 12 Univ. Lyon, UCBL1, INSERM U1028, CNRS UMR5292, CRNL, PSYR2, Lyon, France

**Keywords:** Drug consumption rooms, Harm reduction, People who use drugs, Opioids, France

## Abstract

**Background:**

Drug consumption rooms (DCRs) have been developed in cities with open drug scenes, with the aim to reduce drug-related harm. In Lyon, France's second-largest city, there is no distinct drug use area, which raised doubts regarding the need for a DCR.

**Methods:**

We conducted a face-to-face survey of 264 people who use drugs (PWUDs), recruited in harm reduction or addiction treatment centers, in the streets or in squats. We assess their willingness to use a DCR, and we collected sociodemographic and medical features. Bivariable comparisons and analyses adjusted for sociodemographic parameters explored the association between willing to use a DCR and other variables, thus providing crude (ORs) and adjusted odds ratios (aORs) and 95% confidence intervals (95% CI).

**Results:**

In total, 193 (73.1%) PWUDs accepted to participate (mean age 38.5 ± 9.3 years; 80.3% men). Among them, 64.2% declared willing to use a DCR. Being treatment-seeker (aOR 0.20, 95% CI [0.08–0.51]; *p* < 0.001) and not living alone (aOR 0.29; 95% CI [0.10–0.86], *p* = 0.025) were negatively associated with willing to use a DCR. By contrast, receiving precarity social insurance (aOR 4.12; 95% CI [1.86–9.14], *p* < 0.001), being seropositive for hepatitis C (aOR 3.60; 95% CI [1.20–10.84], *p* = 0.022), being cannabis user (aOR 2.45; 95% CI [1.01–5.99], *p* = 0.049), and reporting previous problems with residents (aOR 5.99; 95% CI [2.16–16.58], *p* < 0.001) or with the police (aOR = 4.85; 95% CI [1.43–16.39], *p* = 0.011) were positively associated.

**Conclusions:**

PWUDs, especially the most precarious ones, largely supported the opening of a DCR in Lyon, a city with no open drug scene.

**Supplementary Information:**

The online version contains supplementary material available at 10.1186/s12954-023-00887-7.

## Introduction

Drug consumption rooms (DCRs) are professionally supervised healthcare facilities in which people who use drugs (PWUDs) can inject and inhale substances in a safe and non-judgmental environment [1]. DCRs primarily aim to reduce individual drug-related harms, including overdose and transmission of infectious diseases, as well as public nuisance, such as drug-related violence or waste [1]. While the first DCRs opened in the 1980s and were initially largely focused on drug injection practices [2], the scope and missions of DCRs have progressively evolved to sometimes include drug inhalation [3], or to offer various services to PWUDs, such as social assistance or primary care service [4]. Overall, the efficacy and cost-effectiveness of DCRs have been largely demonstrated in previous studies [2, 5], making DCRs an important component of the harm reduction armamentarium.

Nowadays, the total number of DCRs in the world is approximately eighty [2]. France has been quite late in developing harm reduction programs for PWUDs, compared to other European countries, even though the French state has been strengthening such programs since the 2000s [6, 7]. For example, in 2005, the French government launched a nationwide program for implementation of “*centres d’accueil et d’accompagnement à la reduction des risques pour usagers de drogues*” (CAARUD), i.e., harm reduction centers, which offer a large coverage of harm reduction services, including syringe exchange, education programs on overdose prevention and management and on healthier drug use practices in general. However, France has been running behind some other European countries in terms of DCR implementation [8], and it was only in 2016 that an experimentation process was launched by the French Ministry of Health [9, 10]. Since then, two DCRs have opened, one in Strasbourg and another in Paris in 2016, leading to substantial reductions in the occurrence of overdoses, the number of visits to emergency wards, and at-risk practices toward HIV and HCV [11, 12].

Lyon, the second-largest metropolitan area in France with almost 1.4 million inhabitants, is a candidate city for the implementation of a DCR. However, unlike in Paris, there is no real open drug scene in Lyon [13], that is, no place with a high concentration of people who use and deal drugs publicly [14]. Consequently, local authorities questioned whether the common DCR model could fit the specific features of drug use in the public space of Lyon, as it is supposed to be implemented amid the most important place of drug use in the city. One of the main questions raised was about the expectancies and requirements of local PWUDs regarding the access to a DCR in Lyon. The “*Dispositifs Territoriaux de Réduction des Risques: Attentes, Besoins et Opinions des Usagers de drogues de Lyon*”, i.e., “Territorial Facilities for Harm Reduction: Expectancies, Needs, and Opinions of Lyon Drug Users” (TRABOUL) study is a survey conducted in a large sample of PWUDs from Lyon. This study aimed to collect their opinions regarding the possible opening of a DCR in the city, and to determine which sociodemographic characteristics among PWUDs were associated with willingness to use a DCR.

## Materials and methods

### Study location and population

A cross-sectional face-to-face survey was conducted between November 1, 2020 and April 30, 2021 in the two harm reduction centers of Lyon, as well as in six outpatient addiction units in the Lyon urban area, and in one prison addiction unit. Investigators were social workers or physicians belonging to a care or harm reduction unit. Participants had to be PWUDs reporting any recent (i.e., less than six months) illicit drug use, excluding cannabis. Opioids obtained through medical prescriptions, including OAT, were not considered as recent drug use. However, the use of prescription opioids was considered a drug use when products had been purchased on the black market. Interviewees had to attend harm reduction facilities or meet harm reduction teams on the streets or in foster homes. They could be undergoing an addiction treatment or not.

### Study questionnaires

The complete study questionnaire is available in Additional file 1 in its translated English version. Sociodemographic characteristics comprised gender (female, male, or other), age (in years), current occupational activity (yes or no, and if yes, which type), marital status (single, in a relationship, living with family or friends, or other), dependent children (yes or no, and if yes, number of children), housing location (postal code) and type of housing (personal, belonging to family or friends, foster home, squat, or other), and social insurance (yes or no, and if yes, which type).

Specific features of the participants’ medical history were investigated, in particular the declared Human Immunodeficiency Virus (HIV) status (negative, positive, or unknown), declared Hepatitis C Virus (HCV) status (negative, positive, i.e., untreated or cured, or unknown), declared Hepatitis B Virus (HBV) status (negative, positive, cured, vaccinated, or unknown). Participants were also asked to specify the type of drugs used (list in Additional file 1) in the past six months and the administration routes (intravenous, snorting, oral, inhalation, or other), as well as the type(s) of material utilized (syringes, snorting straws, inhaling straws, crack pipes, or others), the frequency of drug use with other people (always, occasionally, or never), frequency of drug use in the streets, in a squat, in public toilets, in a car, in an elevator shaft, or other (never, sometimes, or often, for each item), and past issues with inhabitants, storekeepers, police, clients of stores, bars, restaurants, or other. We differentiated crystal-form cocaine, i.e., crack or freebase, from powder-form cocaine, as the profiles of users may differ [15]. Moreover, we created a pooled variable for consumption in public spaces (e.g., streets, public toilets, or stairwells).

We also assessed drug preparation and consumption, that is, average preparation and use duration (in minutes), average frequency of use (average number of uses in a day), daily time of first and last drug use, past arrest by police during drug use in a public space (yes or no), access to a water place during drug use (yes or no), frequency of material sharing (always, sometimes, or never), frequency of personal material reuse (always, sometimes, or never), usual way for disposing of drug use wastes (trash can, street, bottle, addiction or harm reduction unit, pharmacy), past history of overdose (yes or no, and if yes, number), acquaintance with take-home naloxone (yes or no), past use of an intranasal and/or intramuscular naloxone kit (yes or no for each type), preferred naloxone administration route (intramuscular, intranasal, no preference or no opinion).

Another series of questions pertained to opioid agonist treatment (OAT) and addiction treatment, including place(s) and type(s) of current addiction treatment follow-up (none, addiction unit, general practitioner, psychiatrist, psychologist, or other), origin of drug use materials (harm reduction center, addiction unit, automatic dispenser, peer users, internet purchase and postal delivery, or other), current medically-prescribed OAT (none, methadone, buprenorphine, long-acting morphine, or other), frequency of oral OAT use (always, sometimes, never), modes of other OAT use if concerned (injection, snorting, inhalation, or other) and the main personal issue with OATs (limited efficacy, limited access to addiction units, limited access to GPs, issues concerning medical appointments attendance, issues concerning drug use control, or other).

### Variable of interest

Participants were asked to indicate their preferences regarding the opening of additional harm reduction or addiction treatment facilities in Lyon (see Additional file 1; question 35), and more specifically they were asked (question 36.) “*If the creation of a Drug Consumption Room were to take place in Lyon and its metropolitan area, do you think that this facility would be useful to local drug users?*” (yes/no/no opinion) and (question 37) “*Would you yourself use this device today if it existed?”* (yes/no/no opinion).

### Statistical analyses

Categorical variables are presented as numbers and percentages (n, %), while continuous variables are presented as mean and standard deviations (m ± SD). The number of missing values is displayed for each question. We conducted bivariable analyses to determine the associations between willingness to use the DCR as the dependent variable (Question 37 in Additional file 1), i.e., if participants declared that they would use a DCR in Lyon (yes vs. no), and other items as explanatory variables. Since very few people had no opinion about the opening of a DCR, we equated "no opinion" with "no".

Subsequently, logistic regression models were constructed to compare the groups of PWUDs who were willing versus not willing to use a future DCR, thus providing crude (ORs) and adjusted odds ratios (aORs) and their 95% confidence intervals (95% CI). In the multivariable comparisons, clinical parameters were independently used as explanatory variables, while sociodemographic characteristics were used as adjustment variables. Questions related to users’ wishes were not used as explanatory variables as their responses were too closely associated with the dependent variable. Statistical significance is set at *p* < 0.05, unless otherwise specified. Analyses were conducted using the XLSTAT 2022.1.1 software (https://www.xlstat.com/en/).

The study protocol was submitted to the hospital review board which approved it prior to data extraction (CEREVI/2019/003). The procedure was also declared to the *Commission Nationale Informatique et Libertés* (CNIL), in accordance with the French law.

## Results

### Descriptive results

In total, 264 PWUDs (19.2% females; mean age 38.5 ± 9.4 years) were offered to participate in the survey; 193 accepted to be interviewed (response rate 73.1%). Descriptive results of the entire sample can be found in Table 1.

**Table 1:** Descriptive results and bivariable comparisons between PWUDs who were willing versus not willing to use a DCR

Parameter	Total *n* (%)	Will use DCR *n* (%)	Will not use DCR *n* (%)	*p* value
Location of the survey				
Harm reduction center	67 (34.7)	52 (41.9)	15 (21.7)	** < 0.0001**
Addiction unit	91 (47.2)	40 (32.3)	51 (73.9)	
Street	35 (18.1)	32 (25.8)	3 (4.4)	
Gender				
Male	155 (80.3)	101 (81.5)	54 (78.3)	0.612
Female	37 (19.2)	22 (17.7)	15 (21.7)	
Non binary	1 (0.5)	1 (0.8)	0 (0.0)	
Mean age (±SD) (nmv = 3)	38.52 (± 9.35)	38.13 (± 9.67)	39.24 (± 8.81)	0.437
Current activity (nmv = 3)				
Yes	61 (32.1)	33 (27.3)	28 (40.6)	0.059
No	129 (67.9)	88 (72.7)	41 (59.4)	
Marital status (nmv = 1)				
Alone	117 (60.9)	82 (66.7)	35 (50.8)	0.058
In couple	42 (21.9)	25 (20.3)	17 (24.6)	
Living with family/friends/other	33 (17.2)	16 (13.0)	17 (24.6)	
Dependent children (nmv = 3)				
Yes	30 (15.8)	15 (12.4)	15 (21.7)	0.089
No	160 (84.2)	106 (87.6)	54 (78.3)	
Housing location				
Lyon	178 (92.2)	116 (93.6)	62 (89.9)	0.358
Other (Departmental, regional, national)	15 (7.8)	8 (6.4)	7 (10.1)	
Type of housing				
Stable	97 (50.3)	58 (46.8)	39 (56.5)	0.194
Unstable (Street/squat/foster home/friends/family)	96 (49.7)	66 (53.2)	30 (43.5)	
Social insurance for the precarious (nmv = 26)				
Yes	90 (53.9)	71 (66.4)	19 (31.7)	** < 0.0001**
No	77 (46.1)	36 (33.6)	41 (68.3)	
HIV status (nmv = 1)				
Positive	7 (3.7)	6 (4.9)	1 (1.4)	0.394
Negative	174 (90.6)	111 (90.2)	63 (91.3)	
Unknown	11 (5.7)	6 (4.9)	5 (7.3)	
HCV serology status (nmv = 1)				
Positive (untreated and cured)	39 (20.3)	32 (26.0)	7 (10.1)	**0.015**
Negative	132 (68.8)	76 (61.8)	56 (81.2)	
Unknown	21 (10.9)	15 (12.2)	6 (8.7)	
HBV status (nmv = 2)				
Positive	7 (3.7)	4 (3.3)	3 (4.4)	0.062
Negative	141 (73.8)	84 (68.8)	57 (82.6)	
Unknown	43 (22.5)	34 (27.9)	9 (13.0)	
Drug used				
Alcohol	140 (72.5)	93 (75.0)	47 (68.1)	0.304
Tobacco	178 (92.2)	114 (91.9)	64 (92.7)	0.839
Cannabis	144 (74.6)	101 (81.4)	43 (62.3)	**0.003**
Cocaine (crack included)	159 (82.4)	105 (84.7)	54 (78.3)	0.262
Crack-cocaine form	98 (50.8)	72 (73.5)	26 (26.5)	**0.007**
Amphetamine	62 (32.1)	47 (37.9)	15 (21.7)	**0.021**
Heroin	97 (50.3)	60 (48.4)	37 (53.6)	0.486
Morphine	57 (29.5)	45 (36.3)	12 (17.4)	**0.006**
Benzodiazepine	95 (49.2)	68 (54.8)	27 (39.1)	**0.036**
Pregabalin	9 (4.7)	6 (4.8)	3 (4.3)	0.877
Methylphenidate	2 (1.0)	1 (0.8)	1 (1.4)	0.673
Cathinones	12 (6.2)	6 (4.8)	6 (8.7)	0.288
Methadone (without prescription)	35 (18.1)	26 (21.0)	9 (13.0)	0.171
Buprenorphine (without prescription)	35 (18.1)	29 (23.4)	6 (8.7)	**0.011**
Other: ketamine, mushrooms, LSD…	22 (11.4)	15 (12.1)	7 (10.1)	0.683
Materials utilized				
Syringes	59 (30.6)	46 (37.1)	13 (18.8)	**0.008**
Snorting straws	100 (51.8)	54 (43.5)	46 (66.7)	**0.002**
Crack pipes	49 (25.4)	36 (29.0)	13 (18.8)	0.119
Other	22 (11.4)	12 (9.7)	10 (14.5)	0.313
Consumption habits				
Always with partners	160 (83.3)	106 (86.2)	54 (78.3)	0.158
Always in a precarious place	33 (17.1)	21 (16.9)	12 (17.4)	0.936
Occasional in a precarious place	168 (87.0)	111 (89.5)	57 (82.6)	0.171
In public space (i.e., street, toilets, and stairwell)	138 (71.5)	99 (71.7)	39 (28.3)	**0.001**
Problems experienced with				
Residents, shopkeepers, customers… (nmv = 34)	63 (39.6)	54 (52.9)	9 (15.8)	** < 0.0001**
Police (mmv = 34)	43 (27.0)	36 (35.3)	7 (12.3)	**0.002**
PWUDs perceived as being a nuisance (nmv = 67)				
Yes	60 (47.6)	42 (57.5)	18 (34.0)	**0.009**
No	66 (52.4)	31 (42.5)	35 (66.0)	
Drug preparation and drug use habits				
Mean time (min) to prepare (±SD) (nmv = 4)	6.00 (± 4.62)	6.34 (± 4.48)	5.41 (± 4.82)	0.180
Mean time (min) to consume (±SD) (nmv = 13)	21.32 (± 65.23)	17.54 (± 51.08)	28.00 (± 84.75)	0.303
Number of consumptions per day (±SD) (nmv = 18)	5.11 (± 4.58)	4.81 (± 4.17)	5.62 (± 5.18)	0.256
Start time (hour) of consumption (±SD) (nmv = 30)	11.50 (± 4.47)	11.47 (± 4.27)	11.56 (± 4.84)	0.903
Finish time (hour) of consumption (±SD) (nmv = 35)	13.57 (± 9.45)	14.09 (± 9.52)	12.64 (± 9.34)	0.360
Police intervention during drug use (nmv = 7)				
Yes	59 (31.7)	49 (40.8)	10 (15.1)	** < 0.001**
No	127 (68.3)	71 (59.2)	56 (84.9)	
Access to a water point (nmv = 9)				
Yes	70 (38.0)	35 (30.2)	35 (51.5)	**0.004**
Sometimes	72 (39.2)	47 (40.5)	25 (36.7)	
No	42 (22.8)	34 (29.3)	8 (11.8)	
Loan of materials (nmv = 2)				
Yes	47 (24.6)	36 (29.5)	11 (15.9)	**0.037**
No	144 (75.4)	86 (70.5)	58 (84.1)	
Reuses materials (nmv = 1)				
Yes	148 (77.1)	102 (82.9)	46 (66.7)	**0.010**
No	44 (22.9)	21 (17.1)	23 (33.3)	
Fate of the material				
Clean disposal (container, pharmacy…)	71 (36.8)	57 (46.0)	14 (20.3)	** < 0.001**
Inappropriate disposal (street, garbage can…)	122 (63.2)	67 (54.0)	55 (79.7)	
About opioid overdose				
History of overdose	56 (29.0)	39 (31.4)	17 (24.6)	0.317
Number of overdose (±SD) (nmv = 2)	54 (96.4)	2.08 (± 1.77)	1.76 (± 1.09)	0.501
Knows about THN	126 (65.6)	86 (69.9)	40 (58.0)	0.094
Ever used intranasal THN (nmv = 3)	16 (8.4)	9 (7.3)	7 (10.4)	0.458
Ever used intramuscular THN (nmv = 7)	4 (2.2)	4 (3.3)	0 (0.0)	0.138
THN preferences (nmv = 9)				
Intranasal	53 (28.8)	30 (25.6)	23 (34.3)	0.434
Intramuscular	19 (10.3)	12 (10.3)	7 (10.5)	
No matter	112 (60.9)	75 (64.1)	37 (55.2)	
Treated by OAT				
Yes	127 (65.8)	82 (66.1)	45 (65.2)	0.898
No	66 (34.2)	42 (33.9)	24 (34.8)	
Type of OAT prescribed (nmv = 2)				
Buprenorphine	60 (48.0)	44 (55.0)	16 (35.6)	0.055
Methadone	60 (48.0)	32 (40.0)	28 (62.2)	
Morphine	5 (4.0)	4 (5.0)	1 (2.2)	
Routes of OAT administration (nmv = 1)				
Only those authorized	71 (56.4)	39 (47.6)	32 (72.7)	**0.007**
Diverted (snorted, injected, both)	55 (43.6)	43 (52.4)	12 (27.3)	
Diverted routes of OAT administration (nmv = 1)				
Injected	35 (63.6)	29 (67.4)	6 (50.0)	0.534
Snorted	17 (30.9)	12 (27.9)	5 (41.7)	
Both	3 (5.5)	2 (4.7)	1 (8.3)	
Main obstacle for OAT use (nmv = 9)				
Inappropriate treatment	52 (28.2)	39 (32.3)	13 (20.6)	**0.023**
Difficult access to an addiction center	32 (17.4)	27 (22.3)	5 (7.9)	
Difficult access to doctor	29 (15.8)	13 (10.7)	16 (25.4)	
Difficulty in following up	20 (10.9)	13 (10.7)	7 (11.1)	
Difficulties related to other consumption	8 (4.3)	4 (3.3)	4 (6.4)	
Nothing	18 (9.8)	11 (9.1)	7 (11.1)	
Other/not concerned	25 (13.6)	14 (11.6)	11 (17.5)	
Places of consultation (nmv = 4)				
None	37 (19.6)	27 (22.3)	10 (14.7)	**0.045**
Addiction center	114 (60.3)	65 (53.7)	49 (72.1)	
Office based	38 (20.1)	29 (24.0)	9 (13.2)	
Users' wishes				
New addiction center (nmv = 13)	135 (75.0)	83 (73.4)	52 (77.6)	0.533
New harm reduction center (nmv = 13)	147 (81.7)	98 (87.5)	49 (72.1)	**0.009**
A low threshold methadone bus (nmv = 17)	135 (76.7)	89 (80.2)	46 (70.8)	0.154
Drug consumption rooms (nmv = 3)	165 (86.8)	119 (97.5)	46 (67.6)	** < 0.0001**
New automatic syringe dispenser (nmv = 17)	144 (81.8)	95 (88.0)	49 (72.1)	**0.008**
New hospital-based withdrawal service (nmv = 22)	150 (87.7)	88 (84.6)	62 (92.5)	0.123
More psychiatric consultations (nmv = 17)	131 (74.4)	78 (72.2)	53 (77.9)	0.397
More hosting solutions (nmv = 12)	156 (86.2)	106 (93.8)	50 (73.5)	** < 0.001**

*p*-values <0.05 are shown in bold

*DCR* drug consumption room, *HCV* hepatitis C virus, *HBV* hepatitis B virus, *HIV* human immunodeficiency viruses, *MA* marketing authorization, *nmv* number of missing values, *PWUDs* people who use drugs, *OAT* Opioid agonist treatment, *THN* take home naloxone, *SD* standard deviation

Overall, 47.2% of the interviewees were recruited in addiction care units, 34.7% from harm reduction centers, i.e., harm reduction centers, while the remaining 18.1% were recruited in the streets/squats. Men made up 80.3% of the study population; the average age of participants was 38.5 ± 9.3 years. Regarding the participants’ social situation, 67.9% of them were unemployed, 60.9% were single, and 84.2% had no children. In addition, 50.3% of the PWUDs interviewed had a personal housing, while 13.5% had no social security coverage and 53.9% of those who benefited from social insurance were covered by the French specific scheme for precarious people. Regarding viral contaminations to HIV, HCV and HBV, 3.7%, 20.3% and 3.7% declared being positive, and 5.7%, 10.9% and 22.5% declared ignoring their viral status, respectively. Among participants, 18.1% reported taking unprescribed OAT while 65.8% declared receiving a prescription of an OAT. Among those with a prescribed OAT, approximately the same proportion received methadone or buprenorphine. Only 56.4% of them respected the prescribed mode of administration and were not misusing. Among those who reported misuse, injection represented 63.6% of the misuse while snorting represented 30.9%.

The main drug use reported were tobacco (92.2%), cocaine (crack included) (82.4%), cannabis (74.6%), alcohol (72.5%) and heroin (50.3%). All substances pooled, interviewees reported taking an average of 6.0 ± 4.6 min to prepare their drug and 21.3 ± 65.2 min to consume their drug, with an average of 5.1 ± 4.6 episodes of drug use per day. Concerning the equipment used, 51.8% of PWUDs used a snorting equipment, while 30.6% used syringes, and 25.4% pipes. Only 38% reported always having a water point when using drugs. Furthermore, 77.1% of PWUDs declared reusing their drug use material and 24.6% sharing it. Among participants, 63.2% reported disposing of materials inappropriately, including in public space. A majority (83.3%) of PWUDs who used cocaine or heroin did it in groups, and 87% consumed drugs at least occasionally in precarious places. 31.7% of them declared having experienced problems with police forces during a drug use episode, and 39.6% with local residents, shopkeepers, or customers. 47.6% declared that they felt perceived as a nuisance in the public space.

### Factors associated with DCR use

In total, 124 (64.2%) of the 193 interviewed PWUDs reported being willing to use a DCR if one was opened in the city, while 59 (30.6%) of them reported being not willing to use a DCR, and 10 (5.2%) had no opinion. Results of the bivariable comparisons exploring the willingness to use a DCR are presented in Table 1. Compared to participants who did not intend to use the DCR, those who did benefited more frequently from the French special social security coverage for precarious people (66,4% vs. 31.7%; *p* < 0.0001), reported more frequent use of cannabis (81.4% vs. 62.3%; *p* = 0.003), crack (73.5% vs. 26.5%; *p* = 0.007), amphetamines (37.9% vs. 21.7%; *p* = 0.021), morphine (36.3% vs. 17.4%; *p* = 0.006), benzodiazepines (54.8% vs. 39.1%, *p* = 0.036) and non-prescribed buprenorphine (23.4% vs. 8.7%; *p* = 0.011). They injected drugs more frequently (37.1% vs. 18.8%; *p* = 0.008), consumed more in the public space (71.7% vs. 28.3%; *p* = 0.001) but snorted drugs less frequently (43.5% vs. 66.7%; *p* = 0.002), and more frequently declared to share (29.5% vs. 15.9%; *p* = 0.037) and reuse (82.9% vs. 66.7%; *p* = 0.010) their drug use equipment. However, they were more concerned about safely disposing of their dirty drug use materials (46.0% vs. 20.3%; *p* < 0.001). They also declared more frequently feeling to be a source of nuisances (57.5% vs. 34.0%; *p* = 0.009), and reported more frequent diverted use (i.e., snorting or injecting) of their OAT (52.4% vs. 27.3%; *p* = 0.007).

The results of the multivariable analyses comparing the profile of those who would use, versus those who would not use the DCR, are presented in Table 2. Being recruited in a treatment center was negatively associated with willingness to use a DCR, compared to be recruited in a harm reduction center (aOR = 0.20, 95% CI [0.08–0.51]; *p* < 0.001). Living with family, friends/other were negatively associated with intending to attend a DCR, compared with living alone (aOR = 0.29; 95% CI [0.10–0.86]; *p* = 0.025). By contrast, receiving a social insurance for the precarious (aOR = 4.12; 95% CI [1.86–9.14]; *p* < 0.001), being positive (cured or untreated) for hepatitis C (aOR = 3.60; 95% CI [1.20–10.84]; *p* = 0.022) or being a cannabis user (aOR = 2.45; 95% CI [1.01–5.99]; *p* = 0.049) or crack user (aOR = 2.13; 95% CI [1.02–4.48]; *p* = 0.046), were significantly associated with planning to use a DCR. In addition, we observed that a previous history of problems with residents (aOR = 5.99; 95% CI [2.16–16.58]; *p* < 0.001) or with the police (aOR = 4.85; 95% CI [1.43–16.39]; *p* = 0.011) were also factors positively associated with the intention to use a DCR. Last, a trend of association was found with consuming drug in public space (aOR = 2.10; 95% CI [0.97–4.55]; *p* = 0.061).

**Table 2:** Results of the regression models comparing the PWUDs willing and not willing to use a DCR

Parameter	OR [95% CI]	aOR [95% CI] ^a^
Location of the survey		
Addiction treatment unit (vs. harm reduction center)	0.23 [0.11–0.46]***	0.20 [0.08–0.51]***
Street (vs. harm reduction center)	3.08 [0.83–11.47]^†^	2.60 [0.60–11.24]
Gender (male)	1.27 [0.61–2.66]	1.78 [0.72–4.45]
Age	0.99 [0.96–1.02]	0.99 [0.95–1.03]
Current activity (vs. no)		
Yes	0.55 [0.29–1.03]^†^	1.26 [0.56–2.84]
Marital status (vs. alone)		
In couple	0.63 [0.30–1.31]	0.88 [0.35–2.24]
Living with family/friends/other	0.40 [0.18–0.88]*	0.29 [0.10–0.86]*
Dependent children (vs. no)		
Yes	0.51 [0.23–1.12]^†^	0.42 [0.15–1.13]^†^
Housing location (vs. other)		
Lyon	1.64 [0.57–4.73]	4.38 [0.98–19.58]^†^
Type of housing (vs. stable)		
Unstable (street/squat/foster home)	1.48 [0.82–2.67]	1.93 [0.82–4.53]
Social insurance for the precarious (vs. no)		
Yes	4.26 [2.16–8.36]***	4.12 [1.86–9.14]***
HIV status (vs. negative)		
Positive	3.40 [ 0.40–28.93]	3.37 [0.34–33.54]
Unknown	0.68 [0.20–2.32]	0.49 [0.10–2.33]
HCV serology status (vs. negative)		
Positive (cured or untreated)	3.37 [1.39–8.18]**	3.60 [1.20–10.84]*
Unknown	1.84 [0.67–5.05]	1.55 [0.48–5.03]
HBV status (vs. negative)		
Positive	0.91 [0.19–4.20]	1.49 [0.24–9.38]
Unknown	2.56 [1.14–5.75]*	2.00 [0.78–5.09]
Drug used (vs. no use)		
Alcohol	1.40 [0.73–2.69]	1.39 [0.64–3.04]
Tobacco	0.89 [0.29–2.72]	1.02 [0.25–4.10]
Cannabis	2.65 [1.37–5.16]**	2.45 [1.01–5.99]*
Cocaine (powder or crack)	1.55 [0.72–3.26]	1.49 [0.59–3.76]
Crack-cocaine form only	2.29 [1.25–4.19]**	2.13 [1.02–4.48]*
Amphetamine	2.20 [1.12–4.33]*	2.18 [0.89–5.33]^†^
Heroin	0.81 [0.45–1.46]	1.05 [0.50–2.17]
Morphine	2.71 [1.31–5.57]**	1.85 [0.75–4.54]
Benzodiazepine	1.89 [1.04–3.44]*	2.04 [0.96–4.32]^†^
Methadone (without prescription)	1.77 [0.78–4.03]	1.36 [0.44–4.27]
Buprenorphine (without prescription)	3.20 [1.26–8.16]*	2.58 [0.86–7.75]^†^
Materials utilized (vs. no use)		
Syringes	2.54 [1.25–5.14]**	1.78 [0.78–4.07]
Snorting straws	0.39 [0.21–0.71]**	0.33 [0.15–0.71]**
Crack pipes	1.76 [0.86–3.61]	1.64 [0.69–3.90]
Other	0.63 [0.26–1.55]	0.45 [0.15–1.38]
Consumption habits (vs. no use)		
Always with partners	1.73 [0.80–3.73]	1.65 [0.66–4.14]
Always in a precarious place	0.97 [ 0.44–2.11]	0.90 [0.31–2.64]
Occasional in a precarious place	1.80 [0.77–4.19]	1.93 [0.71–5.31]
In public space (street, toilets, stairwell)	3.05 [1.59–5.82]***	2.10 [0.97–4.55]^†^
Problems experienced with (vs. no problem)		
Residents, shopkeepers, customers…	6.00 [2.67–13.50]***	5.99 |2.16–16.58]***
Police	3.90 [1.60–9.48]**	4.85 [1.43–16.39]*
PWUDs perceived as a nuisance (vs. no)		
Yes	2.63 [1.26–5.49]**	3.50 [1.38–8.91]**
Police intervention during a consumption (vs. no)		
Yes	3.86 [1.80–8.31]***	3.15 [1.19–8.33]*
Access to a water point (vs. no)		
Yes	0.23 [0.10–0.58]**	0.14 [0.04–0.49]**
Sometimes	0.44 [0.18–1.10]^†^	0.30 [0.09–0.98]*
Loan of materials (vs. no)		
Yes	2.21 [1.04–4.69]*	1.80 [0.70–4.66]
Reuses materials (vs. no)		
Yes	2.43 [1.22–4.82]*	2.40 [0.98–5.83]^†^
Fate of the materials (vs. inappropriate disposal)		
Clean disposal (container, pharmacy…)	3.34 [1.68–6.63]***	2.81 [1.21–6.48]*
About opioid overdose		
History of overdose	1.40 [0.72–2.73]	1.70 [0.71- 4.06]
Treated by OAT (vs. no)		
Yes	1.04 [0.56–1.94]	1.05 [0.47–2.35]
Type of OAT prescribed (vs. no OAT prescribed)		
Buprenorphine	1.50 [0.70–3.20]	1.68 [0.62–4.55]
Methadone	0.62 [0.30–1.27]	0.65 [0.27–1.59]
Morphine	2.18 [0.23–20.64]	2.21 [0.16–31.61]
Routes of OAT administration (vs. diverted)		
Only those authorized	0.34 [0.15–0.75]**	0.43 [ 0.17–1.12]^†^
Diverted routes of OAT administration (vs. injected)		
Snorted	0.50 [0.13–1.94]	1.45 [0.22–9.74]
Usual place of consultation for OAT (vs. none)		
Addiction treatment unit	0.49 [0.22–1.11]^†^	0.41 [0.13–1.26]
Office based General Practitioner	1.19 [0.42–3.38]	0.70 [0.18–2.72]

*p*-values <0.05 are shown in bold

*DCR* drug consumption room, *HCV* hepatitis C virus, *HBV* hepatitis B virus, *HIV* human immunodeficiency viruses, *MA* marketing authorization, *PWUDs* people who use drugs, *OAT* Opioid agonist treatment, *OR* odds ratio, *THN* take home naloxone, *aOR* adjusted OR (^a^ adjusted for age, gender, current activity, marital status, dependent children, type of housing and social insurance for the precarious), *95% CI* 95% confidence interval

^†^*p* < 0.1; *p < 0.05; **p < 0.01; ***p < 0.001

## Discussion

The main objective of our study was to assess whether PWUDs would be willing to use a DCR in Lyon, France, and to explore the factors associated with the use of such facility. Overall, our study found that almost two-thirds of the PWUDs surveyed would be willing to use a DCR if it was opened in the city. To our knowledge, very few French studies had ever assessed whether PWUDs would be interested in using a DCR. A survey conducted prior to the opening of the Paris DCR included 156 participants, but only 30 were PWUDs [16]. In addition, another study was conducted from the COSINUS cohort survey with a subsample focused on Marseille among 195 participants, a city without a DCR found that more than half (57%) of participants who were ready to use a DCR [17], compared to three-quarters (73%) in our survey conducted in another city without a DCR.

Regarding the main results of our study, we found that almost two-thirds of the interviewees were willing to use a potential DCR in Lyon. The results obtained from our survey conducted in Lyon are higher compared to other French studies [17] and quite similar or below compared to those found in similar international surveys, which found a range between 68.5% and 89.0 [18–22]. A possible explanation for our lower rate could be that part of our sample consisted of treatment-seeking PWUDs who were recruited in treatment centers, whereas other surveys recruited only PWUDs who were not seeking treatment. This hypothesis is supported by the results of the multivariable analyses, which show that participants recruited in an addiction treatment center unit were significantly less willing to use a DCR than those recruited in a harm reduction center. Despite these main limitations, our results are relatively in line with national and, when applicable, international findings on the main points, even if international comparisons should remain careful, as, in other surveys, the target population was more people who inject drugs than PWUDs [21–23].

Similarly, features of precariousness and social isolation, including living alone or benefiting from the social security coverage for precarious people, were also predictive of intending to use a DCR. This is particularly important, insofar as some DCRs offer referral to health and social services [1], and may thus participate in the overall improvement of the health and social conditions of PWUDs.

Past or current HCV contamination, as well as drug injection practices, public space consumption and material sharing habits, were associated with an increased intention to use a DCR. Moreover, having experienced problems with police forces or with residents were also associated with being willing to use a DCR. This might be related to the fact that DCR implementation also implies a memorandum of understanding with local police to avoid clients having adverse police interactions in and around the DCR.

A notable last finding was that crack use was significantly associated with being willing to use a DCR, while heroin use was not. A possible explanation for this finding could be that France currently faces an increasing crack problem, in particular in the most precarious populations [23, 24], which are those who are the most attracted by using a DCR.

All these findings are consistent with recent literature, which found that DCRs aim to attract the most marginalized fringe of PWUDs, i.e., the most precarious ones and those actively using drugs in the public space [1, 2]. This suggests that, in Lyon, as in other cities where DCRs have opened or are planned to be opened, a substantial proportion of PWUDs would benefit from such type of services. A more original finding of our survey was that a substantial part of the PWUDs surveyed were correctly handling their used materials; it is thus possible that a DCR was perceived as a useful means to achieve a clean handling of waste related to drug use. Overall, our results are in line with similar surveys, as we found that the PWUDs willing to attend a DCR were the most precarious people [21, 22], as it is one of the main aims of DCRs to offer such the public a venue for safer and quieter drug use [1, 2].

Our survey had several limitations. The sample combined treatment-seeking and non-treatment-seeking PWUDs was a strategy of recruitment differing from many similar studies, in which only non-treatment-seeking PWUDs were interviewed. Since treatment-seeking PWUDs are less likely to use a DCR, our main results may thus have been biased against the use of a DCR, but, despite this, it remained clear that a large majority of PWUDs were willing to use such a facility. A second limitation was that we did not interview PWUDs who could not answer the questionnaire, in particular those whose command of the French language was insufficient. Although it is difficult to assess the proportion of non-French-speaking PWUDs in Lyon, we can assume that a substantial part of local PWUDs could have been overlooked, with their opinion not taken into account. A last limitation is that it would have been interesting to add a qualitative study on the needs, obstacles and levers for the use of a DCR in Lyon, as it has been done in another studies.

In Lyon, approximately two-thirds of local PWUDs declared willingness to use a DCR if such a facility was to be opened. The most interested PWUDs were those not seeking treatment, living alone, receiving a social insurance benefit for precarious people, seropositive for HCV, using cannabis or crack, and reporting previous problems with residents or police forces. These findings are in line with previous similar studies, but we also show that DCRs attract the most marginalized and precarious PWUDs even in a city such as Lyon, in which street drug use is widespread and not really concentrated in specific open scenes.

### Supplementary Information

**Additional file 1.** Blank form of the survey questionnaire (translated into English).

## Data Availability

Data are available on demand (please contact mathieu.chappuy@chu-lyon.fr).
