# Supplementary material for: Willingness to use a drug consumption room among people who use drugs in Lyon, France, a city with no open scene of drug use (the TRABOUL survey)
Source: Harm Reduct J. 2023 Oct 16;20:149. doi: 10.1186/s12954-023-00887-7 (PMC10580523; doi:10.1186/s12954-023-00887-7)
Supplement: Supplementary file 1 — Additional file 1. Blank form of the survey questionnaire (translated into English). [file 12954_2023_887_MOESM1_ESM.docx]

**English translation for publication.**

**Territorial Harm Reduction Facilities: Expectations, Needs and Opinions of Drug Users in Lyon Metropole**

**TRABOUL STUDY**

**Inclusion criteria: Any user of illicit substances (excluding cannabis exclusively) and/or psychotropic drugs injected, snorted or smoked in the last 6 months.**

**Center carrying out the questionnaire**

# ❒ 1. name of the structure ❒ in the facilities ❒ outside the facilities

# ❒ 2. name of the structure ❒ in the facilities ❒ outside the facilities

# ❒ 3. name of the structure

# ❒ 2. name of the structure

# ❒ 4. name of the structure

# ❒ 5. name of the structure

# ❒ 6. name of the structure

# ❒ 7. name of the structure

# ❒ 8. name of the structure

**Questionnaire number:**

# DCR QUESTIONNAIRE LYON METROPOLE

**1- Are you**

## ❒ a male ❒ a female ❒ other way to define yourself (specify): …………………

1. **Your age (years)**:

1. **Are you currently employed?**

❒ yes, specify : _ _ _ _ _ _ _ _ _ _ _ _ _ _ _ _ _ _ _ _ _ ❒ no

**4- What is your lifestyle?**

❒ alone ❒ in couple ❒ with a family member/relative

❒ other (specify): _ _ _ _ _ _ _ _ _ _ _ _ _ _ _ _ _ _ _ _ _

**5- Do you have any minor child(ren) to support?**

❒ yes, how many : _ _ _ ❒ no

1. **In which district of Lyon/suburb/city outside Lyon do you live?**

_ _ _ _ _ _ _ _ _ _ _ _ _ _ _ _ _ _ _ _ _

1. **What is your accommodation type?**

❒ personal housing ❒ family home/friend's house ❒ home/care center

## ❒ squat ❒ other (specify): _ _ _ _ _ _ _ _ _ _ _ _ _ _ _ _ _ _ _ _ _

**8- Do you have health insurance?**

❒ yes ❒ no

If yes, which one :

❒ Standard with private insurance ❒ Precarious n°1 ❒ Precarious n°2 ❒Serious pathology full coverage

**9- What is your Human Immunodeficiency Virus (HIV) status?**

❒ Negative ❒ Positive ❒ Unknown

**10- What is your Hepatitis C Virus (HCV) status?**

❒ Negative ❒ Positive (active or remitted/cured) ❒ Unknown

**11- What is your Hepatitis B Virus (HBV) status?**

❒ Negative ❒ Positive (active or remitted/cured) ❒ Unknown

1. **Products consumed and consumption patterns in the last 6 months (check all that apply)**

|  | **Intravenous route** | **Snorted** | **Oral route** | **Inhalation** | **Other (specify)** |
| --- | --- | --- | --- | --- | --- |
| **Alcohol** |  |  |  |  |  |
| **Tobacco** |  |  |  |  |  |
| **Cannabis** |  |  |  |  |  |
| **Cocaine** |  |  |  |  |  |
| **Crack/Cocaine based** |  |  |  |  |  |
| **Amphetamines** |  |  |  |  |  |
| **Heroin** |  |  |  |  |  |
| **Morphine (french specialty names)** |  |  |  |  |  |
| **Benzodiazepine (french specialty names)** |  |  |  |  |  |
| **Pregabalin** |  |  |  |  |  |
| **Memthylphenidate** |  |  |  |  |  |
| **Cathinones (3MMC, 4MEC,...)** |  |  |  |  |  |
| **Méehadone without prescription** |  |  |  |  |  |
| **Buprenorphine without prescription** |  |  |  |  |  |
| **Other (specify) :** |  |  |  |  |  |

1. **What type of equipment do you use for your consumption: (several answers possible)**

❒ Syringe ❒ snorting straw ❒ crack pipes ❒ other :

1. **Do you sometimes consume with others?**

## ❒ Always ❒ Sometimes ❒ Never

1. **Do you ever consume in :**

## **Street:** ❒ Always ❒ Sometimes ❒ Never

- **Squat:** ❒ Always ❒ Sometimes ❒ Never
- **Public toilets:** ❒ Always ❒ Sometimes ❒ Never
- **Car:** ❒ Always ❒ Sometimes ❒ Never
- **Stairwell:** ❒ Always ❒ Sometimes ❒ Never
- **Other (specify):** ❒ Always ❒ Sometimes ❒ Never

1. **If yes, have you encountered any difficulties, problems with :**

❒ Residents ❒ Shopkeepers ❒ Police ❒ Customers ❒ Other (specify) : _ _ _ _ _ _ _ _ _ _ ❒ no problem

**17 If you happen to consume in the public space, do you think that your consumption creates or may have created a nuisance in the environment for :**

❒ Residents ❒ Shopkeepers ❒ passers-by in the street ❒ Customers ❒ Other (specify) : _ _ _ _ _ _ _ _ _ _ ❒ no problem

**18 On average, how much time (in minutes) do you need :**

To prepare a product?: _ _ _ _ _ _ _ _ _

## To consume it?: _ _ _ _ _ _ _ _ _ _ _ _ _

1. **On average, how many times do you consume in a day?**: _ _ _ _ _ _ _ _

1. **In general, what is the time:**

- - Of your first consumption of the day?: _ _ _ _ _ _ _ _ _

- - Of your last consumption of the day?: _ _ _ _ _ _ _ _ _ _

1. **Have you ever been stopped by the police while using drugs in public places?**

❒ Yes ❒ No

1. **When you consume, do you have access to a water point?**

❒ Always ❒ Sometimes ❒ Never

1. **Do you ever loan your materials?**

- Yes ❒ No
- **syringe/needle:** ❒ Always ❒ Sometimes ❒ Never
- **cup/gamelle:** ❒ Always ❒ Sometimes ❒ Never
- **filter/cotton:** ❒ Always ❒ Sometimes ❒ Never
- **pipe/tips:** ❒ Always ❒ Sometimes ❒ Never
- **snorting straw:** ❒ Always ❒ Sometimes ❒ Never
- **Other (specify):** ❒ Always ❒ Sometimes ❒ Never

1. **Do you ever reuse your materials?**

❒ Yes ❒ No

- **syringe/needle:** ❒ Always ❒ Sometimes ❒ Never
- **cup/gamelle:** ❒ Always ❒ Sometimes ❒ Never
- **filter/cotton:** ❒ Always ❒ Sometimes ❒ Never
- **pipe/tips:** ❒ Always ❒ Sometimes ❒ Never
- **snorting straw:** ❒ Always ❒ Sometimes ❒ Never
- **Other (specify):** ❒ Always ❒ Sometimes ❒ Never

1. **Where do you usually dispose of your used equipment?**

❒ in a garbage can ❒ in the street ❒ you bring it to an addiction center ❒ in a bottle/can❒ in a needle collector ❒ you take it to a pharmacy

1. **Have you ever overdosed?**

❒ Yes, how many times?_ _ _ _ _ _ ❒ No

**27 Are you familiar with Naloxone (French specialty names of take home naloxone ) ?**

❒ Yes ❒ No

1. **Have you ever used Naloxone at least once, for yourself or for someone you know?**

- **Intranasal form** (French specialty names): ❒ Yes ❒ No
- **Intramuscular form** (French specialty name)**:** ❒ Yes ❒ No

1. **Do you have a preference on the type of Naloxone to use in case of overdose?**

❒ Intranasal ❒ Intramuscular ❒ No matter

**30 Are you currently taking an opioid agonist treatment (OAT) prescribed by a physician**

❒ Yes ❒ No

**If yes, which one?** ❒ Methadone ❒ Buprenorphine ❒ Morphine (with prescription) ❒ Other (specify) : _ _ _ _ _ _

1. **Do you take your OAT by mouth?**

❒ Always ❒ Sometimes ❒ Never

**If other answers than always:**

- - injected ❒ snorted ❒ smoked ❒ other (specify) : _ _ _ _ _ _ _ _ _ _ _ _ _ _ _ _ _ _ _ _ _

1. **What do you consider to be the main difficulty with opioid agonist treatment (one answer possible)?**

❒ Inappropriate treatment (limited efficacy, dosage, taste, mode of administration, etc.)

❒ Difficult access to an addiction center

❒ Difficult access to doctor

❒ Difficulty in following up

❒ Difficulties related to other consumption

❒ Nothing

❒ Other (specify)/not concerned: _ _ _ _ _ _ _ _ _ _

**33 For your care concerning addictions, you consult (several answers possible):**

❒ None ❒ Addiction center ❒ GP’s office ❒ Psychiatry office ❒ Psychology office

❒ Other (specify): _ _ _ _ _ _

**34 Where do you get your consumer materials (multiple responses possible)?**

❒ Harm reduction center ❒ Addiction center ❒ automatic dispensers ❒ pharmacy ❒ through relatives/friends

❒ by postal or direct delivery ❒ Other (specify): _ _ _ _ _ _

1. **Indicate your wishes to open a new facility by completing the table..**

|  | **Extremely important** | **Very important** | **Relatively important** | **Not important** | **Not important at all** |
| --- | --- | --- | --- | --- | --- |
| **New addiction center** |  |  |  |  |  |
| **New harm reduction center** |  |  |  |  |  |
| **A low threshold methadone bus** |  |  |  |  |  |
| **Drug consumption rooms** |  |  |  |  |  |
| **New automatic syringe dispenser** |  |  |  |  |  |
| **New hospital-based withdrawal service** |  |  |  |  |  |
| **More psychiatric consultations** |  |  |  |  |  |
| **More hosting solutions** |  |  |  |  |  |
| **Other (specify) :** |  |  |  |  |  |

1. **If the creation of a Drug Consumption Room were to take place in Lyon and its metropolitan area, do you think that this facility would be useful to local drug users?**

❒ Yes ❒ No ❒ No opinion

1. **Would you yourself use this device today if it existed?**

❒ Yes ❒ No ❒ No opinion

1. **In which district(s) of Lyon and its agglomeration would you like this structure to be located**

❒ Specify: _ _ _ _ _ _ _ _ _ _ _ _ _ _ _ _ _ _ _ _ _

## ❒ No opinion
